# Introduction of the Rapid Deployment Aortic Valve System Use in Elderly Patients With Endocarditis

**DOI:** 10.3389/fcvm.2022.774189

**Published:** 2022-03-22

**Authors:** Alper Öner, Christoph Hemmer, Anthony Alozie, Benjamin Löser, Pascal M. Dohmen

**Affiliations:** ^1^Department of Cardiology, Heart Center Rostock, University of Rostock, Rostock, Germany; ^2^Department of Tropical Medicine and Infectious Diseases, University of Rostock, Rostock, Germany; ^3^Department of Cardiac Surgery, Heart Center Rostock, University of Rostock, Rostock, Germany; ^4^Department of Anesthesiology and Intensive Care Medicine, University of Rostock, Rostock, Germany; ^5^Department of Cardiothoracic Surgery, Faculty of Health Science, University of the Free State, Bloemfontein, South Africa

**Keywords:** active infective endocarditis, rapid-deployment valve system, heart valve surgery, aortic valve, aortic valve endocarditis

## Abstract

**Introduction:**

The rapid-deployment valve system (RDVS) was introduced to facility minimally invasive aortic valve replacement. In this study we evaluate the potential benefits of RDVS in elderly high-risk patients with endocarditis of the aortic valve.

**Materials and Methods:**

Since the introduction of RDVS in our institution in December 2017 through October 2021, EDWARDS INTUITY rapid-deployment prosthesis (Model 8300A, Edwards Lifesciences, Irvine, CA, USA) has been implanted in a total of 115 patients for different indications by a single surgeon. Out of one-hundred and fifteen cases of RDVS implantation, seven patients with a median age of 77 yrs. (range 62–84yrs.), suffered from active infective endocarditis of the aortic valve. The median EuroSCORE II of these highly selected patient cohort was 77% (range 19–80%). Patient data were evaluated perioperatively including intra-operative data as well as in-hospital morbidity/mortality and follow-up after discharge from hospital.

**Results:**

Three patients underwent previous cardiac surgery. Concomitant procedures were performed in six patients including, ascending aorta replacement (*n* = 3), mitral valve repair (*n* = 1), pulmonary valve replacement (*n* = 1), bypass surgery (*n* = 1), left atrial appendix resection (*n* = 1) and anterior mitral valve repair (*n* = 1). Median aortic cross-clamp and cardiopulmonary bypass time was 56 min (range 29–122 min) and 81 min (range 45–162 min.), respectively. Post-operative complications in these elderly high-risk patients were atrial fibrillation (*n* = 3) and re-exploration for pericardial effusion (*n* = 1). One pacemaker implantation was required on postoperative day 6 due to sick sinus syndrome. There was one in-hospital death (14%) and one during follow-up (14%).

**Conclusion:**

Rapid-deployment aortic valve system seems to be a viable option with acceptable morbidity and mortality in elderly high-risk patients with active infective endocarditis of the aortic valve.

## Introduction

Active infective aortic valve endocarditis (AI-AVE) is still associated with high morbidity and mortality, especially in the elderly and multimorbid patients (1–5).

Early surgery in addition to immediate appropriate antimicrobial therapy was proposed in these elderly AI-AVE patients to reduce mortality and embolic events (6, 7). In the study by Lalani et al. (8) early surgery was associated with lower in-hospital and 1-year mortality in the unadjusted analysis and after controlling for treatment selection bias. However, these results could not be replicated after adjustment for survivor bias. The subgroup analysis indicated a lower in-hospital mortality with early surgery in the highest (fifth) surgical propensity quintile. At one year follow-up the lower mortality associated with early surgery was retained both in the fourth and fifth quintiles of surgery propensity group (8). All these indicate an urgent need for further investigations into the effects and timing of surgery in infective endocarditis in patient with indication for surgery.

Another important tool that has been associated with reduction of mortality in patients with AI-AVE is strict implementation of multidisciplinary approach as reported by Botelho-Nevers et al. (9). Due to the highly significant reduction in mortality, this important tool has recently been incorporated into the published European Society of Cardiology guidelines (10).

Regardless of these impressive results, the implementation of surgery recommendations has been suffered a significant setback due to non-referral of patients for surgery. In this context Iung et al. (11) reported that although these guidelines were available and surgery was recommended in 75% of the patients with active infective endocarditis, only half of the patients were operated upon. Prohibitive operative risk due to general status of the patients was cited as reason for non-referral for surgery in 62% of the cases.

New operative techniques are required for the increasing number of elderly patients in need of surgery, including those patients suffering from transcatheter aortic valve endocarditis (12, 13). Sutureless or rapid-deployment aortic bioprostheses were introduced to increase implementation of minimally invasive surgery for aortic valve replacement (MIS-AVR) making it simpler and faster, thereby reducing surgery time and need for blood transfusion, which ultimately facilitates faster recovery and improved survival (14–16). In this context, indications for survival advantage with the use of sutureless bioprostheses in high-risk patients over transcatheter aortic valve intervention has been previously demonstrated by some studies (17–19). Currently, use of sutureless aortic valves are not only limited to MIS-AVR but also in combined cardiac procedures due to shorter implantation time (20–22). This study aimed to evaluate the implementation of rapid-deployment bioprostheses in elderly high-risk patients with AI-AVE.

## Materials and Methods

Between December 2017 and October 2021, 115 patients were treated with EDWARDS INTUITY rapid-deployment prosthesis (Model 8300A, Edwards Lifesciences, Irvine, CA, USA) for different indications by a single surgeon. According to the modified Duke criteria, seven patients were identified with AI-AVE (23). Data were prospectively collected and approved by the local ethical committee. In-hospital mortality was defined as death occurring within 30-days of surgery.

### Patient's Characteristics

**Figure 1:**
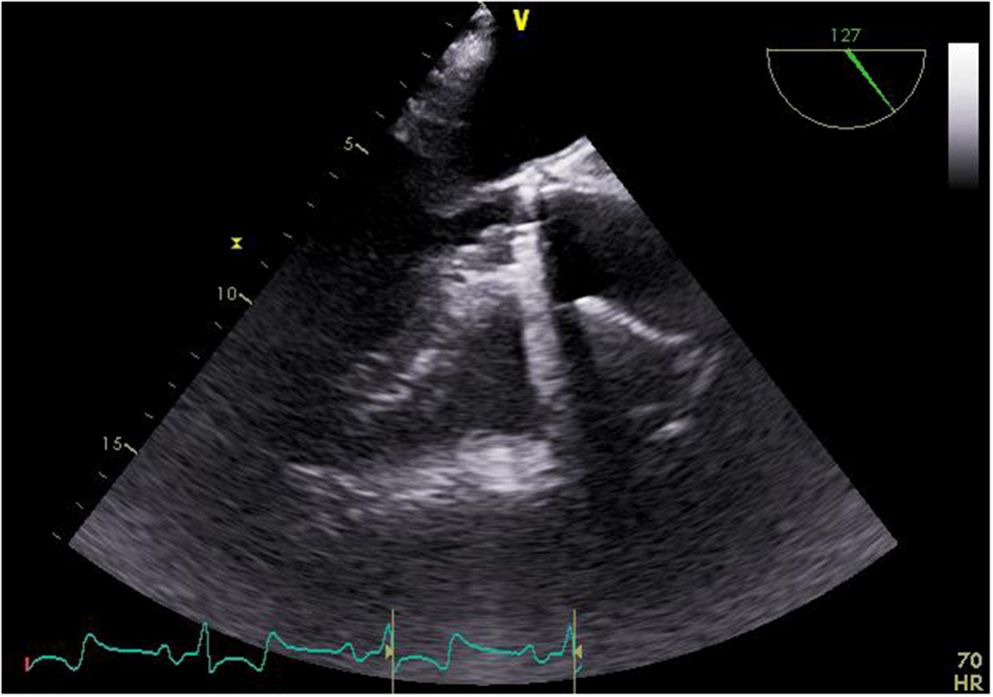
Echocardiographic finding of the vegetation at the annulus of the implanted valve prosthesis.

**Figure 2:**
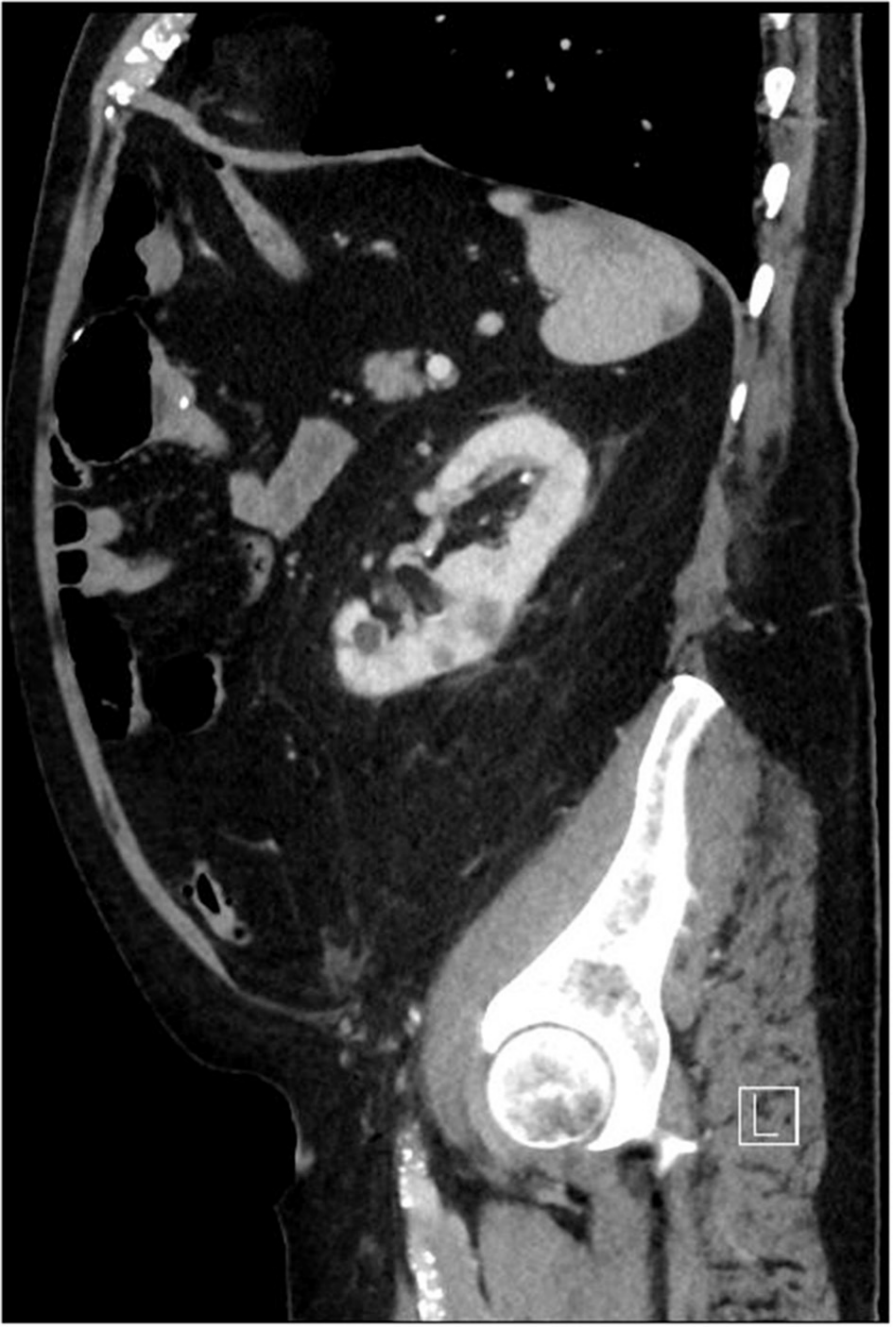
Computed tomography of the chest and abdomen showing septic embolism of the spleen and kidneys.

The median age of the studied patients was 77 years (range 62–84 years). Six patients were males, one patient female. Essential pre-operative characteristics are summarized in Table 1. The predicted mortality was calculated using the EuroSCORE II (median 77%; range 32–80 %). Three patients had moderate pulmonary hypertension (30–55 mm Hg). The pathogen was known in all patients except one, namely *Staphylococcus epidermidis* (*n* = 2), *Aggregatibacter aphrophilus* (*n* = 1), *Rothia dentocariosa* (*n* = 1), *Enterococcus faecalis* (*n* = 1) and *Streptococcus salivarius* (*n* = 1), which were identified by blood cultures. Adequate antimicrobial therapy was initiated in all patients preoperatively followed by regular controls of the infective parameters alongside serial echocardiography evaluations (Figure 1). Despite these measures, all seven patients experienced clinical deterioration and fulfill the modified Duke criteria, warranting consultation of our endocarditis team. Following the team's recommendation of expedited surgery, informed consent was obtained from patient and relatives. Preoperative whole body computed tomography was performed in all patients to identify the entry site for endocarditis as well as previous embolization (Figure 2). Surgical debridement was then scheduled and carried out successfully in all seven patients.

**Table 1:** Pre-operative clinical characteristics.

**Factors**			
Age (y)		77	(62–84)
Male gender		6	(86)
NYHA class III-IV Critical preoperative status Hyperlipidaemia		7 3 4	(100) (29) (57)
Arterial hypertension		7	(100)
Pulmonary hypertension		3	(43)
COPD		4	(57)
Previous cardiac surgery Recent acute myocardial infarction		3 2	(43) (29)
Peripheral artery disease		4	(57)
Chronic renal failure		5	(71)
IDDM		1	(14)
LV ejection fraction (%)		50	(30-55)
EuroSCORE II (%)		77	(32-80)

*Continuous data are presented as median (range) and categorical data as n (%). BMI, body mass index; COPD, chronic obstructive pulmonary disease; IDDM, insulin depending diabetes mellitus; LV, left ventricular; EuroSCORE, European system for cardiac operative risk evaluation*.

### Surgery Details

**Figure 3:**
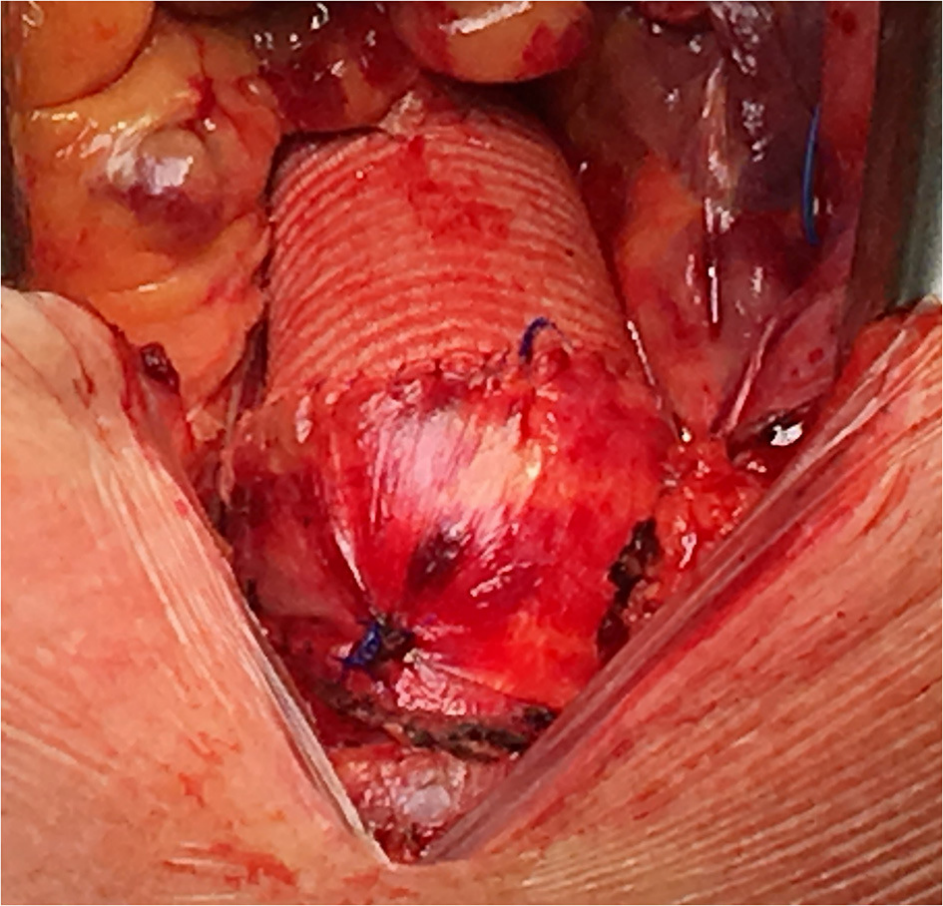
Post-operative finding after aortic valve implantation and ascending aorta replacement.

A RDVS was implanted successfully in all seven patients. The implantation technique has been extensively described in the past (24). The average implanted valve size was 25 mm (range 23–27 mm). Additional procedures were performed in six patients including, ascending aortic replacement (*n* = 3) (Figure 3), mitral valve repair (*n* = 1), pulmonary valve replacement (*n* = 1), bypass surgery (*n* = 1), left atrial appendix resection (*n* = 1) and anterior mitral leaflet repair (Table 2). Median aortic cross-clamping time was 56 min (range 29–122 min) and median cardiopulmonary bypass time was 81 min (range 45–162 min).

**Table 2:** Surgical details.

**Patient no**.	**Concomitant procedure**	**Previous cardiac surgery**	**CC time (min)**	**CPB time (min)**	**Op time (min)**	**Complications**
1	None	No	29	45	135	Pacemaker implantation
2	AAR and anterior mitral valve leaflet repair	No	52	69	173	Pericardial effusion
3	Mitral valve repair	No	66	81	194	Atrial fibrillation, respiratory failure, died
4	Re-pulmonary valve replacement	Ross operation	122	162	341	Atrial fibrillation
5	Re-AAR	Bentall-operation for TAAD	56	138	227	Acute renal failure
6	Single bypass surgery, LAA	Single bypass surgery, LAA	39	70	158	Multi-organ failure, died
7	AAR	AVR and ACB	57	84	239	no

*AAR, ascending aorta replacement; ACB, aortocoronary bypass; AVR, aortic valve replacement; CA, circulatory arrest; CC, cross clamping; CPB, cardiopulmonary bypass; LAA, left atrial appendix; LV, left ventricle; min, minutes; no, number; Op, operation; TAAD, Type-A aortic dissection*.

**Figure 4:**
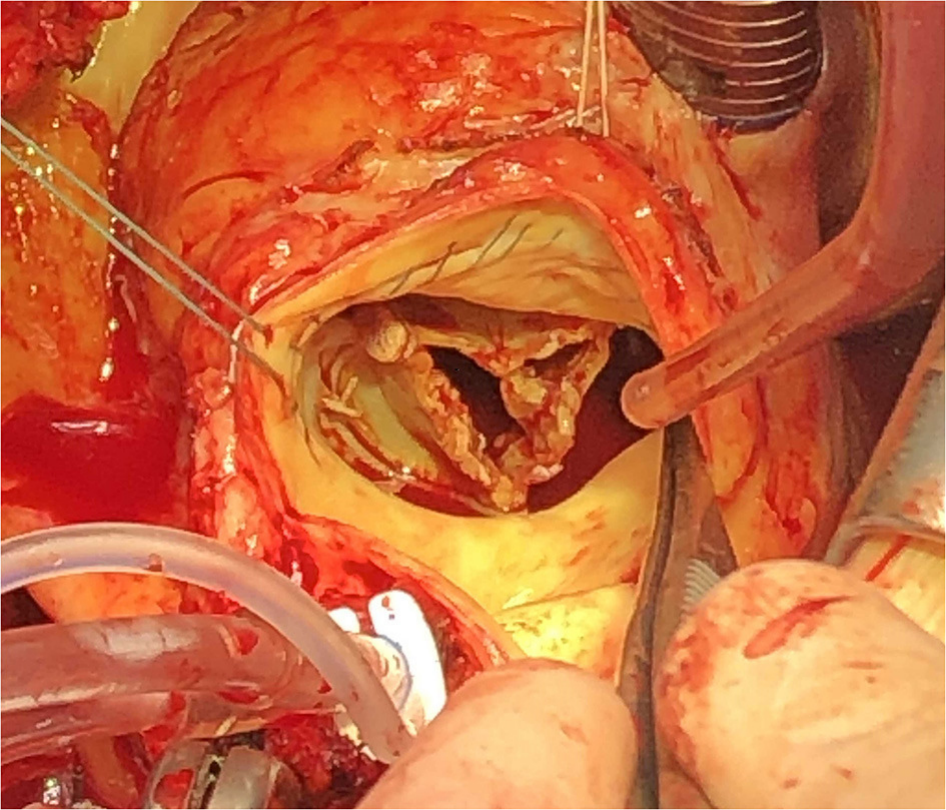
Intraoperative finding of the vegetations at a bioprosthesis.

Three patients underwent re-aortic valve replacement due to infective endocarditis of the aortic bioprosthesis (Figure 4). In one patient, previous surgery was due to type-A aortic dissection, treated with a Bentall-procedure and partial arch replacement. Only this patient was cannulated peripheral using the left femoral vein and right subclavian artery. One patient suffered from pulmonary and aortic valve endocarditis after a Ross procedure. The third patient was re-operated on the aortic bioprosthesis after previous triple coronary bypass surgery with aortic valve replacement. At the time of surgery, all bypasses were patent. An additional aneurysm of the ascending aorta was also treated. On the native and prosthetic valves, large vegetations were noticed in all explants as identified by transesophageal echocardiography. The annulus were in all patients intact and endocarditis was limited to the native aortic leaflets or the valve prosthesis. There were no abscess seen in any of these treated patients.

Intra-operative transesophageal echocardiography showed absence of para- and transvalvular leak in all patients on the end of surgery.

## Results

There was one in-hospital death and one patient died during follow-up. This patient had an initially uneventful postoperative follow-up, without recurrence of endocarditis. He died during follow-up due to respiratory failure. The other patient died of multi-organ failure as sepsis could not be controlled under appropriate antibiotic therapy. One patient was re-explored for pericardial effusion. Another patient required permanent pacemaker due to sick sinus syndrome on day 6 post-surgery. One patient developed acute renal failure which was managed conservatively until recovery of renal function prior to discharge from the hospital. The median intensive-care unit stay was 1 day (range, 1–4 days). Echocardiographic evaluation at discharge demonstrated absence of central- or paravalvular leak with correct position of the rapid-deployment aortic bioprostheses. The average median pressure gradient was 8 mm Hg (rang 5–14 mm Hg). During hospitalization, interdisciplinary examination was undertaken to evaluate clinical and hemodynamics of each patient. All patients received a 6 week antibiogram-guided antimicrobial therapy in accordance with treatment of infective endocarditis guidelines. At median follow-up period of 29 months (range 1–47 months), there was no incidence of re-operation, reinfection, structural/non-structural prosthetic dysfunction, thrombosis, embolism or bleeding events.

## Discussion

Active infective endocarditis remains a uncommon serious disease with considerable morbidity and mortality. Given the proper indications, surgery together with adequate antibiotic therapy can cure the infective pathology of the cardiac tissue and should be comprehensively implemented. Indication for surgery include: cardiac abscess and failure to improve appropriate antibiotic therapy. In older patients, this equally indicates a substantial increase in risk of adverse perioperative outcome (25, 26).

Regardless of the heart valve pathology, recent improvements in conventional valve replacement have demonstrated promise in minimizing cardiac operative risk, especially in high-risk patients. In the aortic position, this includes percutaneous valve implantations as well as sutureless valve prosthesis, and rapid-deployment valve prosthesis (27–29).

The rapid-deployment valve system demonstrated significantly shorter aortic cross-clamp and cardiopulmonary bypass times, which should have a positive effect on morbidity and mortality also in older high-risk patients. Moriggia et al. (30) compared traditional aortic valve surgery with RDVS, demonstrated significant shorter cross-clamp and cardiopulmonary bypass times in patients undergoing full sternotomy. This makes such valve prosthesis even more appealing in special situations as redo-surgeries, combined procedures and high-risk patient unsuitable for transcatheter aortic valve replacement. Moreover, several work groups reported the use of rapid-deployment in special situations such as minimally invasive aortic valve replacement, anomalous coronary arteries, small aortic roots and heavily calcified aortic roots, demonstrating safety and feasibility with potential advantage over conventional aortic valve replacement (31–35).

Another emerging special patient group comprise patients developing endocarditis of the transcatheter aortic valve, as conventional heart surgery was contraindicated in the first place by the heart team. For this reason, experts still debate whether surgery is the treatment of choice in such cases (36, 37). Four reports (38–40) presented such cases, which were treated surgically and showed no 30-day mortality. These findings are further reinforced by the outcome of the results in our series.

In this case series, we present our initial experience with the RDVS in another special situation; active aortic valve endocarditis without abscess formation. We believe conventional aortic valve replacement should be standard for most patients, except in elderly population with prohibitive surgical risk constellation. On the other hand, implementation of sutureless or rapid-deployment valves is quite common in our practice, and the presented patient group comprised highly selected patients with a EuroSCORE II as high as 80%. In patients with additional abscess formation and destruction of the aortic annulus we prefer the self-expandable sutureless aortic valve as previously published (34).

The major postoperative complication encountered was mortality in two cases, which occurred early and later during the postoperative course and was not related to the valve prosthesis. The first patient was multi-morbid, with end-stage renal failure, severe peripheral vascular disease and preoperative stroke. It is noteworthy that transesophageal examination of the aortic valve prosthesis did not reveal valve pathology. The second patients was an octogenarian, which developed multi-organ failure under optimal intensive care support. This mortality rate represents 28% of our “patient population”; however, which we deem acceptable given the very high predicted operative risk, low patient number, and the generally higher mortality in endocarditis patients, who often have chronic renal failure or chronic hemodialysis (11).

Although several studies reported frequent postoperative conduction disorders with need for permanent pacemaker after RDVS, this was not necessary in our patients (25). One patient needed a pacemaker on postoperative day 6, however due to sick sinus syndrome.

One patient needed to be re-explored by pericardial effusion, which is common seen in endocarditis. Youssef et al. found an incidence of 26%, showing a significant correlation in patients by age, left-sided vegetation and splenic infarction/abscesses (41). A similar aspect had our patient.

Although the number of patients presented in this case series is too small to draw a comprehensive conclusion, we have documented encouraging results, especially in terms of the efficacy and safety in the presented patients, who were elderly high-risk patients suffering from acute infection aortic valve endocarditis.

## Conclusion

Rapid-deployment aortic valve prosthesis is effective and practical in surgical treatment in older high-risk patients with aortic valve endocarditis. Available reports provide initial evidence of low morbidity and acceptable mortality, particularly in the elderly high-risk patients.

## Study Limitations

The main limiting factor in this case series the very small number of patients. Our results are in line with those of Lio et al. who also presented a small number (5) of patients (40).

Another limiting factor is the retrospective analysis of the data. To improve the results, prospective studies are encouraged, even though we would discourage from randomization in such cases, so that each patient should get the valve prosthesis that most suits his/her particular anatomical and pathological features.

## Data Availability Statement

The original contributions presented in the study are included in the article/supplementary material, further inquiries can be directed to the corresponding author/s.

## Ethics Statement

The studies involving human participants were reviewed and approved by Ethikkommission an der Medizinischen Fakultät der Universität Rostock. The Ethics Committee waived the requirement of written informed consent for participation.

## Author Contributions

AÖ, CH, AA, BL, and PD drafted and edited the manuscript. All authors contributed to the article and approved the submitted version.

## Conflict of Interest

The authors declare that the research was conducted in the absence of any commercial or financial relationships that could be construed as a potential conflict ofinterest.

## Publisher's Note

All claims expressed in this article are solely those of the authors and do not necessarily represent those of their affiliated organizations, or those of the publisher, the editors and the reviewers. Any product that may be evaluated in this article, or claim that may be made by its manufacturer, is not guaranteed or endorsed by the publisher.
